# Leçons faisant Partie du Cours de Médecine Legale

**Published:** 1821-08

**Authors:** 


### CRITICAL ANALYSES

OF

### RECENT PUBLICATIONS, IN THE DIFFERENT BRANCHES OF MEDICINE AND SURGERY,

### In the Literature of Foreign Nations.

---

Παῖδες ἄρα
Ανδράσιν, ἢ παῖδας ἄνδρες, ἀγαλλόμεθα.

---

*Leçons faisant Partie du Cours de Médecine Legale de* M. Orfila, Professeur de Médecine légale à la Faculté de Médecine de Paris, &c. &c. 8vo. pp. 487.

[In continuation from page 169.]

BARYTES and its *subcarbonate* and *hydrochlorate* (muriate) are rapidly absorbed and carried into the course of the circulation, whether they have been introduced into the stomach, the rectum, or the cavities of the serous membranes, or applied to the subcutaneous cellular tissue or the surfaces of wounds. The accidents which ensue are evidently the results of the absorption of the mineral, and of its influence on the nervous system. It is true that these substances also produce irritation in the parts with which they come in contact; but the almost immediate death produced by them cannot be attributed to that irritation. On from fifteen to twenty grains of either of the preparations above enumerated being applied, diffused or dissolved in water, to a wound in a dog, the animal perishes almost instantaneously.

The principal symptoms of poisoning from this cause, are the following:—an acrid, caustic taste, (from barytes,) or very sharp acrid taste, (from the muriate;) sense of burning in the mouth, pharynx, and the epigastrium; very severe and distressing pains in the epigastric region; nausea; vomiting of mucous and sanguineous fluids, which sometimes will turn green the syrup of violets, (for example, when the poisoning has arisen from barytes, and this substance is present in a large quantity in the matters vomited;) frequent alvine dejections; hiccup; convulsive movements of the muscles of the face, trunk, and limbs; the mouth is sometimes filled with froth; the patient cannot support himself on his limbs, he falls the instant he is placed upright; head-ach and sometimes deafness soon comes on, and the intellectual faculties are disordered. To these symptoms there succeeds, most commonly, considerable depression; the features are then much altered, and death soon takes place.

The *lesions of tissue* are said to be similar to those which result from the caustic irritants.

Ammonia and its *subcarbonate* act on the animal economy like potass and soda, but with more energy, and they more rapidly produce violent convulsions. Inflammation of the mucous membrane of the pharynx and respiratory canals has been produced by their having been held to the nose of persons in the state of syncope. The mu-

6

riate of ammonia, besides the local effects common to the caustic irritants, exerts a deleterious influence on the nervous system and the stomach: indeed, this organ is constantly the seat of more or less intense inflammation, when it has been applied (in considerable quantity) to the subcutaneous cellular tissue, and death has not happened before the lapse of several hours from the time of the application.

The salts and other compounds of *mercury*, *tin*, *arsenic*, *copper*, *antimony*, *silver*, *bismuth*, *gold*, and *zinc*, are classed together.

The *symptoms* of poisoning by those substances are, an acrid metallic taste, more or less analogous to that of ink, and less caustic than that arising from the concentrated acids and alkalies; a sense of constriction in the throat; pain in the mouth, pharynx, stomach, and intestines, at first slight, but soon becoming insupportable; nausea, frequent vomiting of matters of various colours, often mingled with blood, producing no effervescence with chalk, never rendering green the syrup of violets, but sometimes capable of reddening, though in a very slight degree, the tincture of turnstole; when there is diarrhœa, the matters voided from the bowels are sometimes bloody; frequent belchings, often of fetid gases; hiccup; difficulty of respiration, menacing suffocation; the pulse is commonly accelerated, small, contracted, and sometimes irregular and intermittent; there is intolerable thirst, difficulty in voiding the urine, cramps, icy coldness of the extremities; partial or general convulsions; very often great prostration of strength, change of the character of the features, and sometimes delirium.

The *lesions of tissue* from most of the substances under consideration are such as are common to the caustic irritants, only, for the most part, less severe than those from the concentrated acids and caustic alkalies.

The author, in his particular considerations on corrosive sublimate treats of the means of recognizing this substance, under the following several conditions:—1, in the solid state; 2, dissolved in water, alcohol, and ether; 3, mingled with other liquids which have not decomposed it, or decomposed it only partially; 4, united with diverse solid, medicinal substances; 5, combined with liquid or solid aliments which have effected its decomposition; 6, decomposed by the organs of the body, and intimately combined with the tissue of the digestive tube. We shall only adduce his particular observations on the last point, which are as follows.

"If the experiments which have been made with the vomited matters, or with those found in the digestive canal after death, have been insufficient for the discovery of the poison, it will be necessary to seek for it in the tissues themselves. The following fact will show how useful the examination of these parts may become in certain cases of poisoning. If a portion of intestine be macerated in a solution of corrosive sublimate, and the part examined at the end of three days, a considerable portion of the sublimate will be found to have been decomposed and changed into protochloride of mercury, which is intimately combined with the intestine: on this being boiled with distilled water, to free it from the sublimate, not decomposed, on its surface, and then heated with potass in a small alembic, a multitude of globules of mercury will be observed. The result of this experiment shows the necessity of analyzing the portions of the digestive canal on which the poison may have especially acted: this is to be effected by drying them, mixing them with a little potass, and calcining them in an alembic, when the presence of metallic mercury will attest the existence of a mercurial preparation."

Before we quit this subject, we should notice an important case which is likely to occur. A person who has been sick for a considerable time, and habitually constipated, takes, with the intention of purging himself, a few grains of calomel; he dies three or four hours afterwards; it is suspected that he has been poisoned. A medical practitioner is required to examine the body; he finds the digestive tube inflamed; he analyzes the fluids it contains without their furnishing him with any information of the cause of death: he examines the solids, as above advised, and he discovers, at the conclusion of the experiment, a portion of metallic mercury: every thing leads him to believe that there has been poisoning. This opinion is, however, erroneous in the case under consideration; for the redness of the intestinal tube depended on chronic inflammation which the patient had suffered for a long time: the metallic mercury resulted from the dose of calomel he had taken, and which, certainly, could not have produced poisoning. In order to avoid mistakes of this sort, we should know: 1, that calomel which has been introduced into the digestive canal may be found there after death, but that it is, then, most commonly applied to the tissues in the form of a whitish powder, which may be removed by scraping the membranes, because it is not combined with them: besides this, it is insoluble in water, and, when it comes in contact with lime-water at the ordinary temperature, it acquires a blackish colour: it also preserves all its physical properties. If, by chance, it should be intimately mingled with the solid alimentary substances contained in the digestive canal, it would be sufficient to divide these minutely in water: the calomel, from its very considerable specific gravity, would immediately fall to the bottom of the vessel, whilst the other matters would sink only after a longer period. 2. That the calomel which results from the decomposition of corrosive sublimate by vegetable and animal substances, the presence of which is sufficient to enable us to affirm that poisoning has taken place, is never applied in the form of powder to the membranes of the digestive tube; that it never presents itself with its physical properties, because it is intimately combined with the substances which have determined its formation by decomposing the sublimate; lastly, that, if lime-water be poured on the matters thus combined with calomel, no change of their colour can be observed.

The *lesions of tissue* produced by corrosive sublimate are more or less similar to those produced by other irritants, so that it is impossible to ascertain, from the appearance of the dead body, that poisoning from this substance has occurred. However, we should remark, the author observes, that, "in certain circumstances, the tissues on which the corrosive sublimate has been applied are of a whitish-grey colour:

this character, which I have never seen arise from any other poison, is more strongly marked as the quantity of the mineral remaining in the digestive canal after death is greater. Sometimes, also, the internal membrane of the heart is inflamed, and presents here and there spots of a blackish-brown colour."

When corrosive sublimate has been introduced, in the form of powder, into the rectum of a person, directly *after death*, and is left in the intestine for four-and-twenty hours, it produces the following appearances:—The portion of the mucous membrane with which it has been in contact is rugous, or as it were granulous, a little indurated, of a marbly whiteness; and it presents here and there folds of a light rose colour, similar, in their distribution, to venous ramifications. It is sufficient to stretch this membrane by the hand to make these rugosities disappear, and render it quite smooth. The muscular coat corresponding with the mucous membrane above designated, is white as snow: it is the same with the serous tunic, which, besides, presents a remarkable degree of thickening and opacity; the vessels of the meso-rectum are apparently injected; the portion of the intestine with which the poison has not come in contact is in the natural state.

Similar phenomena may be observed, when the corrosive sublimate, reduced to fine powder, has been applied to the rectum an hour and a half after the death of the subject, and when it has been in contact with this intestine for the space of four days. The same appearances are present when two or three ounces of a strong solution of corrosive sublimate has been injected into the rectum three-quarters of an hour after death, and left to act for four-and-twenty hours.

In the case where corrosive sublimate in a pulverulent form has been applied to the rectum, when four-and-twenty hours have elapsed from the death of the subject, we observe, if the body is opened on the ensuing day, that the muscular and serous tunics have become thickened, indurated, and very white, throughout the whole portion of the intestine that has been touched by the poison; the mucous tunic is lined by a greyish matter, interspersed with white spots, and formed of protochloride of mercury and corrosive sublimate. It is impossible to discover the slightest injection of the blood-vessels, or any rose-coloured, or light-red, zones about the intestine.

These observations permit us to draw some inferences of great importance in regard to jurisprudence. It appears from them that it is very easy to determine whether the alterations of tissue produced by corrosive sublimate are the results of the action which it has excited during the life or after the death of the subject; for, besides the characters proper to the lesions occurring under those two conditions, we remark, when the poison has been introduced after death, that the alteration of tissue extends only a little way beyond the portion of intestine with which the poison has been in contact; which is not the case under the other condition, for then there is the appearance of intense inflammation, which decreases gradually as we trace it from some given point, so that there is never a line of demarcation perfectly traced between the parts which are, and those which are not, affected. Besides these circumstances, when the poison has been introduced after death, and in the form of powder, it is found, in considerable quantity, within a short distance from the anus; whilst there is hardly any present when it has been injected during life, the greater part of it having been expelled with the alvine dejections which it has produced.

The author's dissertation on the means of detecting the *oxide of arsenic* under all the conditions in which it may be present in the human body, is an excellent specimen of chemical discussion; but it comprises details too extensive to be transcribed here, and, as it is remarkable for its minute precision rather than any novelty of the processes described, we shall pass over it, as well as his account of the action of this poison on the animal economy, to his observations on the effects of it when introduced into the intestinal canal after death, which are somewhat interesting. When a drachm of oxyde of arsenic, in powder, has been applied to the rectum immediately after the death of the subject, and has been left there for four-and-twenty hours, we observe, on opening the body, that the part of the mucous membrane with which the poison has been in contact is of a very vivid red colour, and presents one or more spots of a blackish-red colour, which are real ecchymoses: the other tunics are in the natural state; it is the same with the portions of intestine which have not been in contact with the metallic oxyd. In the case where it has not been introduced into the large intestine until twenty-four hours after death, we observe, if the body is opened on the ensuing day, that the parts to which the poison has been applied present ecchymoses of various sizes: no other alteration is evident.

Bryony, elaterium, and the rest of the vegetable substances arranged by the author under the head of irritative poisons, are said to produce symptoms which " much resemble" those which have been described as arising from the salts and other compounds of mercury and the rest of the metals just enumerated. For the *lesions of tissue* produced by them, we are referred to what is said of the poisons arranged with the concentrated acids.

On treating of colocynth in particular, the author mentions effects from it of a much more violent kind than we were previously advised of. He says, it results, from several experiments which he has made on animals, and from observations on cases of poisoning in man, that colocynth, in the dose of one or two drachms, is an energetic irritative poison, capable of producing death in the space of four-and-twenty hours, even when it is applied to the cellular tissue of the interior part of the thigh. Its poisonous properties, he says, reside " both in the part which is, and in that which is not, soluble in water."

The gratiola; besides the ordinary symptoms of irritation, " appears to have produced, under certain circumstances, all the accidents of *nymphomania*. (Bouvier, *Gazette de Santé*, 1° Août, 1816.) The women who were the subjects of the cases related by Bouvier, had a soft skin, furnished with very black hair; large prominent veins, strong pulse, great warmth of the extremities, and had been habitually subject to leucorrhœa, hysterical affections, and constipated bowels."

Observations made on the effects of the RHUS RADICANS, (which, according to Mr. Bosc, is a variety of the *rhus toxicodendron*,) lead to the inferences that the most active part of it is that which is disengaged from it in the state of gas, when it does not receive the direct rays of the sun; that it acts in the manner of irritative poisons, and has, besides, a stupefying influence on the nervous system.

The NARCISSUS PSEUDO-NARCISSUS, besides the inflammation which it causes in the organs to which it has been applied, and which in general is not very severe, appears to act especially on the nervous system,—destroying its sensibility,—and on the mucous membrane of the stomach, of which it produces inflammation, even when it has been applied to wounds, or the subcutaneous cellular tissue of the interior part of the thigh; and that its action is more energetic here than when it has been introduced into the digestive canal. The author thinks it "is absorbed and carried into the torrent of the circulation;" whilst, he says, that the juice of the ranunculus acris "appears to be not absorbed." We may mention here, in a general allusion, that we place no reliance on these negative propositions, and are disposed to think the converse inference to be true.

There is nothing else in the author's observations on the rest of the irritative poisons that we can transcribe, conformably with our intentions in this article. We may remark, however, that, excepting in a few instances, where local influence by means of absorption is supposed, he attributes the action of these substances to "inflammation of the organs with which they come in contact, and sympathetic irritation of the nervous system."

The species of FISH arranged in the class of irritative poisons, are *muscles*, the *perca major* of BROWN, the *coracinus fuscus, sparus chrysops, coryphæna hippurus* of LACEPEDE, the *scomber maximus, maroea conger, clupœa thryssa* of LINNÆUS, the *coracinus minor*, and several varieties of *cancer ruricola*. The author advances no opinion of his own, of the reasons why muscles are poisonous under certain circumstances; nor why the other fish, above enumerated, are poisonous from February to July alone, as appears to be the case, according to the observations of Dr. CHISHOLM.

The *narcotic poisons*, according to the author, are such "as act primarily on the nervous system, and on the brain in particular, without producing inflammation of the parts to which they are applied, and which give rise to some one of the following symptoms: torpor, heaviness of the head; somnolency, vertigo, a sort of drunkenness, stupor, a state similar to apoplexy, furious or gay delirium, pains but slight at first, but at length insupportable; plaintive cries, partial or general convulsive movements; weakness or paralysis of the limbs, particularly of the lower ones; dilatation of the pupils; diminished sensibility of the organs of the senses; nausea, vomiting, especially when the narcotic substance has been applied on the ulcerated skin or to the rectum; strong, full, frequent, or slow pulse; respiration but little accelerated, if it be not in the natural state."

When the poisoning terminates in death, "the vessels of the brain and its membranes are often gorged with blood; the lungs are sometimes of a violet colour, or of a more deep-red hue than in the natural state; their tissue is then gorged with blood, and but little crepitant, at least in some parts of them. The blood contained in the cavities of the heart and in the veins does not always preserve its fluidity, as it has been asserted; for it is often found coagulated shortly after death. There is no remarkable lesion of the other organs, and if some degree of inflammation of the digestive tube has been observed after death, it has been produced by some irritating substance which was mingled with the narcotic poison, or it existed before the poisoning."

The narcotic poisons are absorbed and carried into the current of the circulation; and they give rise to the same accidents, whether they have been placed in contact with the ulcerated skin, the subcutaneous cellular texture, the digestive tube, the pleura, the peritoneum, or have been injected into the veins. This uniformity of action is far from being manifest in the greater part of irritative poisons.

We shall cite the author's account of the physical properties of *morphine* and *narcotine*, (the former the purely narcotic, the latter the irritative principle, of opium,) as well as of the tests for those substances, because these may not be so generally known as the properties and qualities of more common matters.

"Morphine is solid, white, or tinged with a yellow or brown colour, according to its degree of purity; its crystals are parallelopipedes; it has no odour. When placed on burning coals, it becomes decomposed in the manner of vegetable substances which do not contain azote, and leaves carbon: when melted in a small glass tube, at a gentle degree of heat, it becomes transparent; but it resumes its opacity as soon as the tube begins to become cold. It is almost insoluble in water; alcohol readily dissolves it when hot, and suffers it to be deposited again to a great extent as it cools. This solution is of a bitter taste, and possesses alkaline properties; it restores the blue colour to tincture of turnsole reddened by an acid. The nitric acid of commerce dropped on morphine, communicates to it a beautiful red colour. It rapidly dissolves in cold acetic acid, and it will combine with all the acids, and form with them crystallizing salts.

"Narcotine is solid, white, or slightly tinted with yellow, inodorous, insipid, and crystallized in straight prisms with a rhomboidal base. When gradually heated in a glass tube, it melts like fat substances, at a slightly elevated temperature, becomes transparent, and preserves this state even after it has again become cold. If the temperature be carried to a higher degree, or if it be placed on burning coals, it becomes decomposed and emits a thick smoke having an ammoniacal odour. It is hardly at all soluble in cold water; boiling alcohol dissolves it very readily, and suffers it to be deposited again on the solution becoming cold. It is very soluble in ether; and oil of olives or almonds dissolves it slowly, at a degree of temperature inferior to that of ebullition. None of these solutions possesses alkaline properties. Acetic acid, whatever may be its degree of concentration, dissolves it only at the boiling heat. The nitric acid of commerce dissolves it when cold, without changing it to a red colour; the solution is yellow.

From the results of experiments made on dogs, and observations on man, relative to the action of morphine on the animal economy, it appears:—"1. That pure morphine, in the solid state, may be introduced into the stomach of the weakest dog, in the dose of ten or twelve grains, without giving rise to any sensible phenomena; which depends on the great difficulty with which the gastric juices dissolve it: but if there were in this viscus a considerable quantity of free acid, the morphine would be dissolved, and would produce all the symptoms of poisoning. 2. That it does not act when applied in the solid state to the subcutaneous cellular tissue of the interior part of the thigh of dogs. 3. That the salts of morphine produce on man the same effects as the aqueous extract of opium: the sulphate and hydrochlorate act with less energy than the acetate. 4. That the action of twelve grains of morphine dissolved in acetic acid is more violent than that of the same quantity of aqueous extract of opium. 5. That morphine dissolved in olive oil exerts a much more energetic action on the animal economy, than the same quantity of aqueous extract of opium: thus, a quantity of oily solution, containing six grains of morphine, is as powerful as twelve grains of the extract. 6. That it is probable, from some observations made on man, that morphine dissolved in alcohol acts with still greater force than the oily solution; but this fact cannot be proved on dogs, because alcohol diluted so as to exert no action on these animals, dissolves such a small quantity of morphine that it is impossible to produce the least apparent effect in them with it. 7. That the soluble preparations of morphine are absorbed; their action is much more violent when they have been injected into the veins, than when they have been applied to the cellular tissue or the digestive tube. 8. It acts on the animal economy like the aqueous extract of opium."

It appears from experiments made on dogs with *narcotine*:—"1. That ten or twelve grains of this substance may be applied to the cellular tissue at the interior part of the thigh, without producing the slightest accident. 2. That ten or twelve grains, dissolved in six or eight drachms of olive oil, and introduced into the stomach, cause the following effects:—fifteen or eighteen hours after the administration, the animal suffers nausea, which is soon followed by vomiting; he appears weakened, and as it were in a state of stupor; his posterior extremities gradually bend; respiration is a little accelerated; soon after this, he rises up and leans forward, and seems more lively. This state continues for several hours, until the animal becomes so weak that he is obliged to lie on his belly or his side; an attitude in which he dies at the end of a few hours. Death is preceded by slight convulsions of the limbs: it takes places at the end of the second, third, or fourth, day; but no vertigo, paralysis of the limbs, plaintive cries, nor strong convulsive shocks, are observed, as is the case from morphine and opium: the sensual organs exercise their functions very freely. On opening the body, we discover no morbid alteration in the digestive canal. 3. That a grain of narcotine dissolved in oil, and injected into the jugular vein, produces a state of stupor analogous to that above described, and may cause death in the space of four-and-twenty hours. 4. That twelve grains dissolved in about two drachms of strong vinegar, may be injected into the cellular texture of the interior part of the thigh, without there resulting from it any perceptible inconvenience; whilst the same quantity of acetate of morphine, applied to the same tissue, gives rise to all the symptoms of poisoning."

The author arranges in the class of *narcotico-acrid poisons* only such as " produce both *narcotism*, and inflammation of the parts with which they come in contact." The author makes several subdivisions of this class; in the first of which he places *squill*, *œnanthe crocata*, *aconite*, *hellebore*, *colchicum*, *belladonna*, *datura stramonium*, *tobacco*, *digitalis*, diverse species of *cicuta*, and the *rose-bay*.

The symptoms produced by these poisons are, " agitation, shrieks, delirium of a vivid kind; convulsion of the muscles of the face, jaw, and limbs; dilatation of the pupils, in a great proportion of cases; the pulse is, in different instances, strong, frequent, and regular, or small, slow, and irregular; more or less acute pains about the epigastrium, and in several parts of the belly; nausea, obstinate vomiting, purging. Sometimes, instead of much agitation, a sort of drunkenness, great depression, insensibility, and general tremor, are observed, when the patients have no inclination to vomit. The whole of the symptoms just described may not be present in the same individual; but those which are manifested never completely cease to re-appear some time afterwards, as happens from the poisons ranged in the two other subdivisions of this class, of which we shall speak hereafter."

" The organs with which those poisons have been in contact for a certain time, are the seat of more or less intense inflammation, similar to that produced by the irritants arranged with the mineral acids. The lungs, the blood, and the brain, present appearances similar to those which are manifest as the effects of the narcotic poisons."

All these poisons are absorbed, and act especially on the brain or some other parts of the nervous system. The inflammation they produce cannot be regarded as the cause of death.

The *œnanthe crocata* produces effects from which may be inferred: " 1. That it should be ranged amongst poisonous substances. 2. That it produces most commonly, when it has been received into the stomach, the following symptoms:—burning heat in the throat and about the epigastrium; cardialgia, diarrhœa, somnolency, vertigo, mental alienation, violent convulsions, a very strong spasmodic action of the muscles of the jaw; the skin sometimes becomes covered with rose-coloured spots, of irregular figure, and which, successively, become enlarged. 3. That it produces more or less intense inflammation in the parts with which it has been in contact. 4. That its deleterious effects appear to depend on its absorption and its action on the nervous system."

We cannot cite any thing remarkably novel from the rest of the particular descriptions of the effects of the poisons of this group: we proceed, therefore, to the second subdivision of narcotico-acrid poisons, which consists of *nux vomica*, *faba sancti ignatii*, *upas tieuté*, and *strychnine*. The symptoms of the effects of these substances, in man and dogs, are, " general uneasiness; contraction of the muscles of the body generally, with which the spinal column is drawn backward; to this contraction, which is of very short duration, there succeeds a state of remarkable calmness, which is followed by a new tetanic paroxysm, which is of much longer duration than the first, and is accompanied with an acceleration of the breathing. These accidents suddenly cease, respiration becomes slower, and the patient appears as it were bewildered. Soon after this, there is another general convulsion; there is then observed in dogs a stiffness of the whole body, the fore-legs are directed backwards, the spine is also bent backwards, and the head lays back on the neck; respiration is very rapid; soon afterwards, there is stiffness and immobility of the hind legs, the chest and the head are thrown upwards; the animal falls down, at first on his lower jaw, and then soon lays on his side: at this period there is complete tetanus, immobility of the thorax, and cessation of respiration. This state of asphyxia, announced also by the violet colour of the tongue and gums, continues for one or two minutes, during which the organs of the senses and the brain continue to exercise their functions; at least, unless the asphyxia be carried to the highest degree, for then the actions of these organs begin to be weakened. The termination of this paroxysm is announced by the sudden disappearance of the tetanus, and by the gradual re-establishment of respiration. Soon afterwards a new attack comes on: the contractions now are still more violent; the convulsive shocks are very strong, and similar to those which would be produced by a current of galvanism directed along the spine of an animal just killed; there is asphyxia again, and convulsions of the muscles of the face. Death happens most frequently at the end of the third, fourth, or fifth paroxym, ordinarily seven or eight minutes after the manifestation of the first symptoms, sometimes later. A circumstance worthy of being remarked, and which is observed only in the effects of the poisons under consideration and in those of the false angustura and brucine, is, that touching any part of the body, menaces, or a sudden noise, readily produce this general tetanic contraction.

Numerous dissections of the bodies of animals poisoned by the substances of this class, have showed the same kind of appearances as in those of persons who have died asphyxiated; and not the slightest trace of inflammation in the digestive canal has ever been observed. The author, however, relates a case in which inflammation of the mucous membrane of the stomach and small intestines appeared to result from the ingestion of a large quantity of nux vomica, in a man.

It appears, from the author's experiments, that the poisons under consideration act with the greatest energy when they have been introduced into the thoracic and abdominal cavities, or into the jugular vein: their action is less powerful when they are applied to the subcutaneous cellular tissue, or injected into arteries remote from the heart; and still less powerful when they are applied to the mucous surfaces; and they seem to exert no influence on animals whose spinal marrow has been removed by thrusting a rod of whalebone through the spinal canal.

The *ticunas*, *woorara*, and *curare*, are said by the author to produce the following effects. The animals submitted to their influence lie in a state of langour; their pulse is hard and frequent, respiration short and quick; the muscles, especially those of the pectoral extremities, are paralyzed after having suffered a convulsive contraction; the body becomes cold, and respiration ceases. He thinks,—in which point he differs from Mr. BRODIE,—that they act on the spinal marrow rather than on the brain: they occasion, he remarks, neither stupor nor abolition of sensibility, but they suspend respiration. Their action differs, he adds, from that of the upas tieuté, in their paralyzing more promptly the voluntary muscles, without exciting such violent and frequent convulsions and spasms; it differs from that of the upas antiar, in not producing either paralysis of the heart or purging.

*Camphor*, and *cocculus indicus* (with its active principle *picrotoxine*,) form the third subdivision of narcotico-acrid poisons.

If one or two drachms of camphor dissolved in an ounce of olive oil, or three or four drachms of cocculus indicus finely powdered, or ten or twelve grains of picrotoxine, be administered to a dog, the animal, after a few minutes, if he has not vomited, (for these substances very often produce severe vomiting,) becomes restless, agitated; his walk is trembling, and the muscles of the face are somewhat convulsed. After the lapse of from five to fifteen or five-and-twenty minutes, he suffers a violent paroxysm, characterized by the following symptoms:—falling down on the side, whilst the head is sometimes strongly drawn backwards; very severe convulsions, especially in the limbs: he pitches backwards, striking the ground with his head with great violence, and his body is rolled about in all directions; the conjunctiva is injected with blood; the eyes are prominent, and insensible to external impressions. The animal now no longer hears; he may be poked and drawn about, and hallooed at, without showing any sensibility: his mouth is filled with a thick foam; his tongue and gums are livid; and respiration is, as it were, suspended. This fit lasts for three or four minutes, and is sometimes terminated by efforts to vomit. The animal then remains for fifteen, twenty, or five-and-twenty minutes, without any sign of disease: he seems to have recovered the use of his senses, and might be supposed to be quite well; but another paroxysm similar to the former, only that it is more violent, soon appears, during which the animal yells in a horrible manner; his respiration is very laborious; and accompanied with the exhalation of a large quantity of vapour having the odour of camphor, when this substance has been administered. This attack, in which the animal most commonly dies, lasts for six or eight minutes: it is often preceded by vertigo, and more or less debility of the anterior extremities; these precursory symptoms are sometimes of eight or ten minutes' duration. Cases of poisoning in man prove that camphor produces effects in him similar to those just described.

If the bodies are opened immediately after the death of the animals, the left ventricle of the heart is found to contain blood of a reddish-brown colour. The heart has finally ceased to act; the lungs are contracted, but little crepitant, more dense than ordinary, and interspersed with strata of a deep colour. The brain presents no remarkable alteration. The digestive canal most frequently shows traces of inflammation, or of ulceration, when the poisoning has been produced by camphor; whilst it has the healthy appearance in cases where death has been produced by cocculus indicus and picrotoxine. The author thinks that death arises from asphyxia; or, at least, from the difficulty with which respiration is effected during the convulsive shocks. He seems to attribute nothing expressly to the state of the heart.

The subjects next considered are the poisonous champignons; which we shall notice, with the rest of the work, in the next Number.

---

### Medical and Physical Intelligence.

